# Should disability-inclusive health be a priority in low-income countries? A case-study from Zimbabwe

**DOI:** 10.1080/16549716.2022.2032929

**Published:** 2022-03-15

**Authors:** Hannah Kuper, Tracey Smythe, Tapiwa Kujinga, Greaterman Chivandire, Simbarashe Rusakaniko

**Affiliations:** a International Centre for Evidence in Disability, London School of Hygiene & Tropical Medicine, London, UK; b Pan-African Treatment Access Movement, Harare, Zimbabwe; c Leonard Cheshire Disability Zimbabwe, Harare, Zimbabwe; d Family Medicine, Global and Public Health Unit, Faculty of Medicine and Health, University of Zimbabwe, Harare, Zimbabwe

**Keywords:** Disability, inclusion, health, Zimbabwe

## Abstract

The National Disability Policy was launched in Zimbabwe in June 2021 and includes a range of commitments for the provision of disability-inclusive health services and rehabilitation. Fulfilment of these pledges is important, as at least 7% of the population have disabilities, and people with disabilities face greater challenges accessing healthcare services and experience worse health outcomes. However, it will require financial investment which is challenging as the needs of people with disabilities are set against a background of widespread health systems failures in Zimbabwe, exacerbated by the COVID-19 pandemic. Zimbabwe currently faces an epidemic of TB and HIV and a growing burden of non-communicable diseases (NCDs) with a lack of investment, healthcare staff or infrastructure to provide the necessary care. Urgent action is therefore needed to strengthen the health system and ‘build back better’ after both the pandemic and the regime change. The Zimbabwean government may face the dilemma, common in many low-resource settings, of whether to focus on disability or to wait until the health system has been strengthened for the majority. This paper proposed four complementary arguments why it is important to focus on people with disabilities. First, this focus respects the rights of people with disabilities, including those specified in the new National Disability Policy. Second, it will be challenging to reach the Sustainable Development Goals, including those on health and other global health targets, without including people with disabilities. Third, there is a growing rationale that disability-inclusive health systems will work better for all, and fourth, that they will create cost savings. Everyone will therefore benefit when the health systems are designed for inclusion. In conclusion, a focus on disability may help to strengthen health systems for all as well as helping to achieve human rights and global development goals.

## Background

The National Disability Policy was launched in Zimbabwe in June 2021 and includes a range of commitments for the provision of disability-inclusive health services and rehabilitation (Box 1) [1]. The aspirations of the policy are comprehensive and ambitious, but fulfilment of these pledges will require financial and strategic investment, including in the health system. These commitments are justified, as at least 7% of the population of Zimbabwe have disabilities, equating to one million people [2]. Furthermore, people with disabilities are at higher risk of morbidity and mortality, including from COVID-19, yet face greater barriers in accessing healthcare [3–5]. However, the needs of people with disabilities are set against a background of widespread health systems failures in Zimbabwe’s once proud health system, following decades of under-investment, mismanagement and hyper-inflation [6]. Health care expenditure is low and declining, falling from 10.5% of GDP in 2010 to 4.7% in 2018 [7]. Meanwhile, Zimbabwe faces an epidemic of TB and HIV and a growing burden of non-communicable diseases (NCDs), while neglected tropical diseases remain endemic (Table 1). COVID-19 has further exposed the fragility of the Zimbabwe health system with one clinician reporting that *‘The health system is on its knees’* [8]. Urgent action is therefore needed to strengthen the health system and ‘build back better’ after both the pandemic and the regime change in 2017. The Zimbabwean government may face the dilemma, common in many low-resource settings, of whether to include a focus on disability or to wait until the health system has been strengthened for the majority. In this paper we will consider the rationale for ensuring that efforts to strengthen health systems in Zimbabwe include people with disabilities.

**Box 1.** Zimbabwe National Disability Policy 2021: Commitments to disability-inclusive health [1]

–Provision of services
∘Ensure access to health services to people with disabilities – both general health services and disability-specific
∘Comprehensive habilitation and rehabilitation services and programmes must be organized, strengthened and extended to persons with disabilities
∘Access to free health services in public health care institutions
∘Provide people with disabilities the same range, quality and standard of health care as provided to other persons.
–Rights and ethics
∘Outlaw discrimination against people with disabilities in health structures
∘Ethical standards must be upheld in treatment of people with disabilities (e.g. provision of free and informed consent to treatment)
–Healthcare workers
∘Aim for 15% of health students to be people with disabilities
∘Health professionals must be trained about disability
–Healthcare settings
∘Healthcare facilities, consultations and public health campaigns should be accessible
∘Services must be locally provided

**Table 1.** Demographic and health system indicators for Zimbabwe, and the neighbouring countries of Botswana, Mozambique, South Africa and Zambia

	Zimbabwe	Botswana	Mozambique	South Africa	Zambia
Socio-demographic profile*					
Population size in 2020 (million)	14.9	2.4	31.3	59.3	18.4
GDP per capita in 2020 (USD)	1,215	6,405	449	5,656	985
HDI rank (out of 189 countries) in 2020	150	100	181	114	146
Adult literacy rate (2013–19)	89%	87%	61%	87%	87%
% of population<$1.90 per day (2014–19)	34%	15%	64%	19%	59%
Effective UHC Indicators (2019)**					
UHC effective coverage index	54	58	44	60	53
Met need for family planning with modern contraception	85	78	47	81	64
Antenatal/peripartum/postnatal care for newborns	19	20	14	20	21
Antenatal/postpartum/postnatal care for mothers	11	32	19	28	25
MCV1 coverage	83	85	97	79	94
DTP3 coverage	82	84	95	63	93
Diarrhoea treatment	92	86	84	71	79
LRI treatment	49	71	50	80	62
ART coverage	94	94	50	81	81
TB treatment	22	66	24	64	51
Breast cancer treatment	24	47	16	42	26
IHD treatment	39	60	51	79	61
Stroke treatment	34	37	27	59	15
Diabetes treatment	24	10	14	21	16
Additional health indicators (2015)***					
Population at risk sleeping under ITB (% <5 population)	15%	31%	73%	-	69%
Households with access to at least basic sanitation %	36%	77%	29%	76%	26%
Health service inputs (2018)*					
Per 1000 population					
–Number nurses/midwives	1.9	5.4	0.7	1.3	1.3
–Number of physicians	0.2	0.5	0.1	0.9	1.2
–Hospital beds	1.7	1.8	0.7	2.3	2.0
% of GDP on health	4.7%	5.9%	8.2%	8.3%	4.9%
Out of pocket health expenditure	24%	3%	10%	8%	10%
Health outcome indicators*					
Life expectancy at birth (2019)	61	70	61	64	64
Maternal mortality (100,000 live births) (2017)	458	144	289	119	213
Neonatal mortality (1000 live births) (2019)	26	18	29	12	23
Infant mortality (1000 live births) (2019)	38	32	55	28	42
HIV prevalence (15–49 years) (2020)	12%	20%	12%	19%	11%
Diabetes prevalence (20–79 years) (2011)	10%	11%	3%	7%	5%
Tuberculosis incidence (100,000 people) (2020)	193	236	368	554	319
Malaria incidence (1000 people at risk) (2018)	51	0.6	305	2	157

Source: *Reference [12]; **Reference [9]; ***Reference [13].

## Current state of the Zimbabwe health system

Zimbabwe is a low-income country, with 34% of its population living on under $1.90 per day (Table 1). Compared to its neighbours, Botswana and South Africa are richer, Zambia is similar in poverty level, while Mozambique fares worse. The Universal Health Coverage (UHC) effective coverage index closely matches poverty across these five countries [9].

Within Zimbabwe, there are some areas for optimism. Coverage of family planning, vaccination, ART and diarrhoea treatment are high. Life expectancy is now 61, up from a nadir of 43 in 2004 [10]. HIV prevalence in adults is approximately 13%, down from a peak of 25% in the mid-1990s [11]. However, these bright spots are overshadowed by the many signs that the health system is failing in Zimbabwe [12]. Antenatal care is low and maternal mortality is twice as high in Zimbabwe (458 per 100,000 live births) as for Zambia (213), and three times as high as Botswana (114) or South Africa (119) [9,12]. There is poor treatment coverage for conditions such as cancer, ischaemic heart disease, stroke, asthma and epilepsy and sanitation coverage remain low [9,13]. Life expectancy is higher only than Mozambique for the region [12]. Strikingly, compared to neighbouring countries, Zimbabwe has few physicians and spends little on health [12]. Out of pocket health expenditure is very high – 24% – more than twice as high as in neighbouring countries [12].

There is therefore a clear narrative in Zimbabwe. Lack of investment in health is reflected in low service level inputs and consequently poor health coverage and outcomes. These overall figures hide great inequalities, and the population living in rural areas is likely to experience even worse healthcare access. Investment in health and scale-up of services is needed, urgently.

## Priorities for health system strengthening in Zimbabwe

The need to strengthen the health system is well-recognised in Zimbabwe. The Strategy for 2020–2025 is still in development [14], but is built upon the recently expired National Health Strategy [15], and is likely to include the following 10 strategic focus areas:

1. Improved access to essential medicines and commodities;
2. Increased access to water, sanitation and healthy environment;
3. Improved health infrastructure and medical equipment for Health Service Delivery;
4. Improved governance of the Health Service;
5. Improved health sector human resources performance;
6. Increased domestic funding for health;
7. Reduced morbidity and mortality due to communicable and NCDs;
8. Improved reproductive, maternal, new-born child and adolescent health and nutrition;
9. Improved public health surveillance and disaster preparedness and response, and
10. Improved primary, secondary, tertiary, quaternary, and quinary care.

These priorities appear appropriate to tackle the key challenges highlighted above. A question therefore remains as to whether a focus on disability now should be a priority, given these stark challenges to the health system. The previous strategy made few commitments to provide disability-inclusive health [15].

## Disability and health in Zimbabwe

The national disability survey from 2013 estimated that approximately 7% of people in Zimbabwe had disabilities, and they were on average poorer (Table 2) [2]. This prevalence is lower than international figures and so is likely to be an underestimate (e.g. 15% global prevalence [3]). The survey uncovered stark health inequities; People with disabilities had worse health, whether assessed in terms of self-reported physical health, mental health, or wellbeing. They also reported a higher prevalence of specific health conditions such HIV, diabetes or cancer, also documented in other African settings [16–20].

**Table 2.** Health status of people with and without disabilities in Zimbabwe: results of the national survey [3]

	Zimbabwe	
	People with disabilities*	People without disabilities
Poverty		
SES Mean score	6.2	6.8
Food insecurity (Sometimes/often no food to eat in household during the last two weeks)	12%	9%
Self-reported health		
% reporting good physical health	58%	92%
% reporting good mental health	70%	95%
Health and wellbeing score (WHODAS)		
–Males	23	28
–Females	22	27
Health conditions		
–HIV and AIDS	10%	5%
–STIs	5%	4%
–Diabetes	4%	2%
–TB	5%	3%
–Cancer	2%	1%
Have information and knowledge about		
–HIV and AIDS	75%	85%
–STI	64%	73%
–Diabetes	56%	61%
–TB	66%	73%
–Cancer	56%	61%
Problems in understanding health information		
–HIV and AIDS	10%	8%
–STIs	9%	7%
–Diabetes	10%	9%
–TB	10%	8%
–Cancer	12%	9%
People with Disabilities Only		
–Environmental barriers in availability of health and medical care (daily/weekly)		
–Males	12%	
–Females	11%	
–Urban	8%	
–Rural	13%	
–Medical rehabilitation		
–Needed	40%	
–Received	19%	
–Satisfaction with service	36%	
–Assistive device		
–Needed	39%	
–Received	14%	
–Satisfaction with service	23%	

*Disability was defined in terms of reporting difficulties in one of the following 6 functional domains: mobility, vision, hearing, communication, remembering/concentrating, self-care [2].

The national survey in Zimbabwe also highlighted informational barriers, as people with disabilities reported having less information and knowledge about different health conditions and greater difficulties understanding the health information being provided [2]. Reports of barriers to accessing healthcare were also frequent among people with disabilities, particularly those living in rural areas. These findings are consistent with the World Report on Disability which found that people with disabilities were two times more likely to find healthcare providers’ skills and facilities inadequate, three times more likely to be denied healthcare and four times more likely to report being treated badly in the healthcare system [3]. People with disabilities are not a homogenous group, and although data are lacking it is likely that some people with disabilities (e.g. those with intellectual impairments) may face particular difficulties in accessing healthcare.

Most healthcare provision can be offered at the primary care level, but many people with disabilities can also benefit from rehabilitation. Here too, the national survey highlighted large gaps. Approximately 4 out of 10 people with disabilities needed medical rehabilitation, but only half had received this service [2]. A similar pattern existed for assistive devices. Moreover, satisfaction with both medical rehabilitation and assistive devices was low; for assistive devices 4 out of 10 people said that their device was not in good working condition. There were frequent reports by people with disabilities that health services were too expensive, and that this presented a barrier to access. Out of pocket payments are also likely to be incurred in procuring assistive devices; 67% of people who had an assistive device reported that they had sourced it privately, and only 33% through government centres.

## Rationale for disability-inclusive health system strengthening

The health system challenges in Zimbabwe are therefore great, amplified further by the COVID-19 pandemic. The Zimbabwean government will need to increase its health spending to address the stark health issues and build back better systems. Nevertheless, there are at least four important arguments for Zimbabwe for maintaining a focus on disability and thereby supporting the implementation of the 2021 Zimbabwe National Disability Policy.

(1) Respecting the rights of people with disabilities

The 2021 Zimbabwe National Disability Policy re-asserts the right to healthcare and rehabilitation of people with disabilities [1]. These rights are also explicitly stated in the UN Convention of the Rights of Persons with Disabilities, which was ratified by Zimbabwe in 2013 [21]. The 2013 Constitution of Zimbabwe and planned National Health System strategy both reinforce the country’s commitment to realising the rights to health for all, implicitly including people with disabilities [14].

(2) Meeting health and other development goals

There is a global commitment to move towards UHC as part of efforts to achieve Sustainable Development Goal 3 to ‘ensure healthy lives and promote well-being for all at all ages’. UHC means that everyone receives the quality health services that they need without suffering financial hardship. Zimbabwe too supports efforts towards UHC – in 2018 the Minister of Health and Child Care stated *‘Our planning is focused on equity in service use, service quality improvement and financial protection for all Zimbabweans’* [22]. The mention of ‘for all’ and ‘equity’ are important, as within UHC, there is a ‘Leave no one behind’ agenda, to ensure that groups such as people with disabilities are included. The limited available evidence presented above shows that people with disabilities in Zimbabwe are falling behind in the core pillars of UHC. Realising SDG targets around UHC and commitments to equity in healthcare will be difficult to meet if this large group continues to be left behind. Good health is also important for inclusion in other aspects of life, such as education and employment, and so failure to provide disability-inclusive healthcare can also hamper efforts to achieve other SDGs.

Achieving other health goals will also be more challenging without a focus on disability. Let us take HIV as an example. UNAIDS has set the 95-95-95 target for 2030 (95% of people with HIV have been diagnosed, of whom 95% are on ART, and 95% of treated people are virally suppressed). Zimbabwe has made remarkable progress in its fight against HIV, since the bleak days of the 1990s (Table 2) [23]. The goal of 95-95-95 by 2030 therefore seems to be within grasp, but these plans could be scuppered through exclusion of people with disabilities. If 25% of people with HIV have disabilities [16], then for a hypothetical group of 1000 people with HIV, 250 will have disabilities and 750 will not (Table 3). Let us assume, relatively conservatively, that people with disabilities in Zimbabwe are 10% less likely to be tested, access treatment or continue on ARTs (to allow viral suppression). As we move along the cascade, it is apparent that outcomes are consistently worse for people with disabilities. Moreover, they make up at least one in three people for whom the target is not met. The closer the goal is to being met (e.g. the % of people on HIV treatment), the greater the proportion of people with disabilities among those for whom the target is not met. Disability inclusion in HIV services is therefore important, which is recognised by the Zimbabwe National HIV and AIDS Strategic Plan (2015–2020) [24].

(3) Building better health systems for the future

**Table 3.** HIV, disability and the achievement of the UNAIDS 95:95:95 goals

Population group	Number	Know HIV status	On HIV treatment	Virally suppressed
Total	1000	900 (90%)	846 (94%)	728 (86%)
Disabled	250	209 (84%)	182 (87%)	145 (80%)
Not disabled	750	691 (92%)	664 (96%)	583 (88%)
Target not met		100	54	118
% disabled		41%	50%	31%

Disability-inclusion does not have to be at odds with providing healthcare for all. In fact, there is a strong rationale that disability-inclusive health systems will be more cost-effective and future-proofed.

The new National Disability Policy priorities for disability-inclusive healthcare focus on improving the availability and accessibility of health services, and training of healthcare workers on disability. These priorities are consistent with the forthcoming National Health Strategy. Disability-inclusion should cost little if included in the design phase (e.g. in training, in building accessible facilities), and will also improve systems for people with chronic conditions and people with other types of diversity. For instance, staff trained to communicate effectively with people with communication impairments may also find it easier to consult with minority language speakers. Training to improve attitudes towards people with disabilities may help overcome stigma to other groups, such as LGBT persons or ethnic minorities. Facilities that are accessible for people with mobility impairments will work better for others, such as people who are older, those with temporary impairments or parents with buggies. Meeting the healthcare needs of people with disabilities will also require scaling up availability of rehabilitation, which is rightly an important priority of the Disability Policy. Rehabilitation will increasingly be needed in Zimbabwe anyway, as the disease burden shifts towards NCDs and the population continues to age.

(4) Creating cost savings for the health systems

Investing in healthcare for people with disabilities will create cost savings to the health system [25]. Equal access to mainstream services, such as HIV prevention, for people with disabilities could save thousands of dollars per person in lifetime treatment costs, as well as reduce the risk of further infections. Improved access to disability-specific services can also be cost-saving as illustrated by clubfoot, a condition in which the structure and position of the foot are affected. The average cost of the Ponseti treatment to correct clubfoot is US$167 per patient. The average number of DALYs averted is 7.42, yielding a cost-effectiveness ratio of US$22.46 per DALY averted [26]. This is less than a tenth of the cost of many other treatment modalities used in resource-poor settings. There is also growing evidence that disability-inclusive healthcare services may increase earnings and labour productivity and increase tax revenue [25,27]. In Zimbabwe, the higher rates of unemployment and labour market inactivity among people with disabilities, combined with reduced productivity of employed people with disabilities have led to a loss of 3.8% GDP (USD 128 million) [28]. Everyone will therefore benefit when the health systems are designed for inclusion.

## Conclusion

A focus on providing disability-inclusive primary healthcare alongside scaling up disability-specific services may help to strengthen Zimbabwe’s health systems for all as well as helping to achieve human rights and global development goals.

## Paper context

The one billion people with disabilities frequently have greater healthcare needs but face many barriers in accessing services. Low income countries such as Zimbabwe face the dilemma of whether to focus on disability-inclusion now or after the health system has been strengthened for the majority. This paper proposes four arguments for why disability should be prioritised now: 1) realization of rights, 2) achievement of health goals, and 3) development of cost-effective and 4) future-proofed health systems.
